# A Ketone Ester Drink Enhances Endurance Exercise Performance in Parkinson’s Disease

**DOI:** 10.3389/fnins.2020.584130

**Published:** 2020-09-30

**Authors:** Nicholas G. Norwitz, David J. Dearlove, Meng Lu, Kieran Clarke, Helen Dawes, Michele T. Hu

**Affiliations:** ^1^Department of Physiology, Anatomy and Genetics, University of Oxford, Oxford, United Kingdom; ^2^Department of Sport and Health Sciences, Oxford Brookes University, Oxford, United Kingdom; ^3^Oxford University Hospitals NHS Foundation Trust, Nuffield Department of Clinical Neurosciences, Oxford, United Kingdom

**Keywords:** β-hydroxybutyrate, BDNF, exercise, ketone ester, Parkinson’s disease

## Abstract

**Objectives:**

Routine exercise is thought to be among the only disease-modifying treatments for Parkinson’s disease; however, patients’ progressive loss of physical ability limits its application. Therefore, we sought to investigate whether a ketone ester drink, which has previously been shown to enhance endurance exercise performance in elite athletes, could also improve performance in persons with Parkinson’s disease.

**Participants:**

14 patients, aged 40–80 years, with Hoehn and Yahr stage 1–2 Parkinson’s disease.

**Intervention:**

A randomized, placebo-controlled, crossover study in which each participant was administered a ketone ester drink or an isocaloric carbohydrate-based control drink on separate occasions prior to engaging in a steady state cycling test at 80 rpm to assess endurance exercise performance.

**Outcomes Measures:**

The primary outcome variable was length of time participants could sustain a therapeutic 80 rpm cadence. Secondary, metabolic outcomes measures included cardiorespiratory parameters as well as serum β-hydroxybutyrate, glucose, and lactate.

**Results:**

The ketone ester increased the time that participants were able to sustain an 80 rpm cycling cadence by 24 ± 9% (*p* = 0.027). Correspondingly, the ketone ester increased β-hydroxybutyrate levels to >3 mmol/L and decreased respiratory exchange ratio, consistent with a shift away from carbohydrate-dependent metabolism.

**Conclusion:**

Ketone ester supplementation improved endurance exercise performance in persons with Parkinson’s disease and may, therefore, be useful as an adjunctive therapy to enhance the effectiveness of exercise treatment for Parkinson’s disease.

## Introduction

Parkinson’s disease (PD) is the second most common neurodegenerative disorder in the world, affecting 1% of adults over the age of 60 and growing in prevalence at an alarming rate (Tysnes and Storstein, 2017; GBD 2016 Parkinson’s Disease Collaborators, 2018). PD is identified pathologically by the death of dopaminergic neurons in the substantia nigra pars compacta (SNpc) of the midbrain, which disrupts subcortical motor loop function and leads to the classic motor symptoms of bradykinesia, tremor, rigidity, and postural instability. There are currently no disease-modifying drug therapies for PD. Standard treatment involves dopamine replacement with levodopa, a medication that has a temporary window of efficacy, can induce dyskinesias, and does not address the burdensome non-motor symptoms of PD (Hinnell and Ray, 2009).

By contrast, moderate to vigorous exercise is thought to be among the only treatments may change the disease’s course. Certainly, it is near impossible to prove that exercise is disease-modifying in PD, as to do so would require large long-term prospective trials in which patients are randomized to exercise therapy or a control. However, the combination of animal model data showing the vigorous exercise is neuroprotective, and retrospective epidemiological studies showing moderate to vigorous exercise is associated with reduced risk, together support the notion that exercise might be a disease-modifying treatment for PD.

Animal models of PD have demonstrated that physical activity is neuroprotective against dopaminergic toxins, such as 6-OHDA and MPTP. For example, rats forced to routinely run on a treadmill shortly after being injected with 6-OHDA exhibited preserved motor function and retained twice as many SNpc dopaminergic neurons as non-exercised controls (Tajiri et al., 2010). Exercise dosage also appears to be important. In an MPTP murine model of PD, 3 months of exercise prior to SNpc lesion protected against dopaminergic neuron and striatal dopamine losses, but not when the exercise was restricted to two-thirds or fewer wheel revolutions (Gerecke et al., 2010). These and other models demonstrate that exercise attenuates motor symptom deficits, in association with the preservation of dopaminergic neurons and/or dopaminergic neurotransmission (Ahlskog, 2011).

The animal literature dovetails with retrospective studies demonstrating vigorous exercise in midlife reduces the risk of developing PD (Chen et al., 2005; Thacker et al., 2008; Xu et al., 2010). In the largest of these studies, exercise history, including both light and moderate to vigorous exercise, was assessed in a population of over 200,000 participants. After adjusting for potentially confounding variables, the study found that moderate to vigorous exercise at midlife was associated with a 38% reduced risk of developing PD. However, no significant reduction in risk was observed for light physical activity. This study also included a meta-analysis of the existing retrospective trials examining the relationship between past physical activity and the development of PD, which all reported reductions in disease risk of similar magnitude (overall 33% reduction in risk) (Xu et al., 2010).

Recently, a phase II randomized clinical trial, the Study in Parkinson’s Disease of Exercise (SPARX), reported that high-intensity treadmill exercise, but not moderate-intensity exercise, prevented progression of motor symptoms over 6 months in newly diagnosed patients. SPARX included 128 participants with recently diagnosed PD who were randomized into high-intensity, moderate-intensity, and sedentary control groups. After 6 months, the high-intensity exercise group exhibited no change in UPDRS motor score, whereas both the moderate intensity and control groups exhibited declines in motor function (Schenkman et al., 2018). The animal data, retrospective epidemiological studies, and SPARX trial all suggest that vigorous exercise is disease-modifying in PD.

How vigorous physical exercise exerts its disease-modifying potential in persons with PD disease is unknown. However, a range of possible mechanisms exist and include exercise-induced increases in the expression of neurotrophic factors, like GDNF and BDNF (Cohen et al., 2003; Sleiman et al., 2016), which are depleted in the PD brain (Chauhan et al., 2001); enhancement of dopaminergic neurotransmission via increased vesicular release of dopamine as well as the restoration of dopamine receptors (Vuckovic et al., 2010; Petzinger et al., 2013); increased VEGF and regional blood flow (Villar-Cheda et al., 2009); or improvements in oxidative stress, inflammation, and immune signaling (Cadet et al., 2003; Petzinger et al., 2013). It is likely that any clinical effect would be a combination of these and other undiscovered mechanisms.

Our study used cycling as a mode of exercise. Prior interventional studies examining the therapeutic effects of cycling exercise in PD have emphasized cadence as the critical variable. Patients who cycle at higher cadences of around 80 revolutions per minute (rpm) exhibited motor benefits that persist for at least a month and are not observed in those who exercise at lower cadences (Ridgel et al., 2009, 2015). For the purposes of this study, we took 80 rpm to be a therapeutic threshold. Unfortunately, the possibility that 80 rpm cycling confers benefits in PD presents a paradox. The progressive nature of the motor disease undermines the long-term efficacy of vigorous exercise as a potentially neuroprotective strategy. The purpose of this study was to explore whether a nutritional supplement could boost exercise performance in PD, thereby better enabling patients to engage in what may be the only available disease-modifying treatment for their condition.

Specifically, we chose to examine whether a ketone ester (KE) supplement, ΔG^®^, could increase patients’ ability to sustain an 80 rpm cycling cadence. KE provides the bioidentical ketone molecule, D-β-hydroxybutyrate (β-HB), to that produced by the human body during periods of carbohydrate scarcity to fuel the human brain and muscles (Norwitz et al., 2019). Furthermore, it is proven safe (Soto-Mota et al., 2019), improved cycling performance in elite athletes (Cox et al., 2016), and, particularly in PD, may help to circumvent the respiratory chain complex I blockade and recover intramuscular β-oxidation (Landin et al., 1974; Saiki et al., 2017; Norwitz et al., 2019). In the discussion, we elaborate up these and other mechanisms of action by which KE could improve exercise performance in PD and explain why the β-HB molecule, which itself has neuroprotective properties in PD, adds to the promise that needed disease modifying treatments for PD are on their way.

## Methods

### Participants and Screening

This study (registered ISRCTN16599164) was approved by external research ethics committees (National Health Service Health Research Authority and South Central – Oxford A Research Ethics Committee, REC reference: 19/SC/0032) and was conducted in accordance with the declaration of Helsinki (2008). Fifteen participants were recruited from the Oxford Parkinson’s Disease Centre (OPDC) Discovery cohort and the study was conducted at Oxford Brookes University, Headington campus, between July and December 2019. Participants were Hoehn and Yahr stages 1–2, between the ages of 40 and 80, non-smokers, and did not possess a condition, other than PD, that would restrict their ability to exercise. Levodopa equivalent daily dose (LEDD) was calculated using methods previously reported (Tomlinson et al., 2010). Participants provided written informed consent prior to inclusion. Following informed consent, but prior to exercise, participants underwent a resting twelve-lead electrocardiogram. One participant was dismissed because of an irregular cardiac rhythm, bringing the total number of recruited and completed participants to *n* = 14. Participant characteristics are shown in Table 1.

**TABLE 1:** Participant characteristics.

Age (years)	62.2 ± 1.5	Male / Female	10/4
Height (meters)	1.70 ± 0.02	Disease duration (years)	4.9 ± 1.0
Weight (kg)	72.4 ± 3.3	Hoehn and Yahr	1–2
BMI (kg/m^2^)	25.1 ± 1.1	Levodopa equivalent dose	520 ± 93

*Values are reported as means ± standard error.*

### Baseline Test

To determine the appropriate fixed wattage at which to set the cycle ergometer for subsequent testing, participants engaged in a baseline test. This test also served as a familiarization session for subjects, allowing them to become accustomed to the laboratory and exercise equipment before the 80 rpm endurance test (below). Participants came into the lab at either 8:00 or 10:00 am fasted (>8 h) and following overnight withdrawal of dopaminergic therapy (levodopa and dopamine agonists), termed “off” medication. After being cleared for exercise by a resting twelve-lead electrocardiogram and Physical Activity Readiness Questionnaire, participants were instructed to cycle 80 rpm for as long as possible on an electronically braked cycle ergometer (Excalibur Sport, Lode Netherlands), integrated with a cardiopulmonary monitoring system (Metalyzer 3B, Cortex, Germany) that controlled the work rate protocol on the ergometer. The test began with a 4-min warmup set at 50 Watts. Thereafter, wattage increased by 10 Watts every 2 min until the participant stated s/he was too tired to continue or could not sustain a cadence of >70 Watts for a cumulative total of 20 s. Resting heart rates, heart rates taken at the end of each step, and approximated maximum heart rate (220 – age) were used to create a linear regression of wattage as a function of heart rate. Each participant’s fixed-Watt value for subsequent tests was set at 55% of their projected wattage at maximum heart rate. In order to standardize test length, some participants’ warmup was set to 25 Watts and some participant’s fixed-Watt value was set at 65% of their projected wattage at maximum heart rate. These adjustments helped to compensate for individual participants’ exercise tolerances for subsequent endurance testing. Absolute load did not impact outcomes and the randomized cross-over design ensured that these necessary personalized adjustments on the baseline test did not impact the integrity of the data.

### Drink Preparation and Randomization

The two drink preparations used in this study were a ketone ester plus carbohydrate (KE+CHO) drink, consisting of 25 mL ΔG^®^ and 45 g dextrose diluted in an additional 25 mL water, and an isocaloric (300 kCal) taste-matched carbohydrate (CHO) drink, consisting of 75 g dextrose and 1.5 mL Symrise bitter flavor (product code: SY648352) dissolved in 50 mL water. Drinks were prepared in a room separate from participants and administered in an opaque container. Their viscosity and taste profiles were matched such that participants could not discriminate between the drinks.

As this was a randomized crossover study, each of the fourteen participants took part in both arms of the study on testing days that were separated by 1 week. The order in which the KE+CHO and CHO drinks were presented was determined by a random number generator, with even numbered participants receiving KE+CHO first and odd numbered participants receiving CHO first. By chance, ten participants received KE+CHO first and four participants received CHO first demonstrating a distribution that did not unfairly bias our results, given the expected learning effect (performance on the second visit was 6 ± 9% better than that of the first, *p* = 0.512) and would only serve to underestimate the effect size of the KE+CHO treatment.

### 80 rpm Endurance Test

To assess the impact of ketone supplementation on patients’ exercise performance, participants came into the lab in an “off” medication and overnight fasted (>8 h) state at either 8:00 or 10:00 am, similar to the baseline testing procedure. At 30 min prior to exercise testing, participants had β-HB, glucose, and lactate levels measured by fingerstick (FreeStyle Precision Neo and Lactate Scout+ blood analyzers). A new lancet was used to draw blood from either the index or middle finger and first drop blood was discarded prior to measurements. Immediately thereafter, participants received either the KE+CHO drink or the taste-matched CHO drink.

**FIGURE 1:**
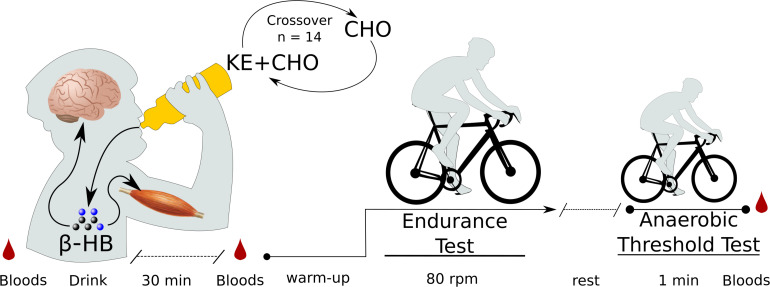
Exercise test protocol. *N* = 14 participants came into the lab on two separate testing days on which they received either a ketone ester plus carbohydrate (KE+CHO) or an isocaloric carbohydrate (CHO) drink in a randomized crossover fashion. Before drink consumption, before exercise (30 min after drink consumption), and following exercise, bloods were measured by fingerpick. Exercise testing occurred on a fixed-Watt cycle ergometer. Exercise began with a 4-min warmup, after which wattage was increased to each participant’s personalized fixed wattage set by baseline testing. For the endurance test, participants cycled at 80 rpm until failure. For the anaerobic threshold test, participants cycled at as high a cadence as possible for 1 min.

Thirty minutes after receiving the study drink (KE+CHO or CHO), participants again had β-HB, glucose, and lactate levels measured before beginning the test. This time point was chosen based on previous data showing that the KE given at a similar dose to healthy participants reaches peak blood levels at between 30 and 60 min (Stubbs et al., 2017). The beginning of this time window was selected as a measuring point such that participants would have either stable or still increasing β-HB levels when they began exercise testing and maximum β-HB would be available for oxidation. The endurance test was conducted on the same electronically braked cycle ergometer and began with the same 4-min warmup as in the baseline test, before the power was raised to each participant’s personalized fixed wattage. Participants were verbally encouraged to maintain 80 rpm until failure, defined as the point at which the participant could not sustain a cadence of >70 Watts for a cumulative total of 20 s or s/he chose to stop (Figure 1).

In addition to the primary variable of interest, endurance performance (the duration of time participants could sustain 80 rpm), we also measured ventilation rate (V_E_), oxygen consumption rate (VO_2_), VO_2Peak_, carbon dioxide exhalation rate (VCO_2_), and respiratory exchange ratio (RER), calibrated to room O_2_ and CO_2_ concentrations, using the Metalyzer 3B cardiopulmonary monitoring system. We carefully distinguish VO_2Peak_ (the highest VO_2_ obtained during the endurance test) from the standard nomenclature, VO_2Max_, because the latter definitionally necessitates an increasing exercise load, which was not possible given the target primary outcome variable and parameters of this study design. Nevertheless, VO_2Peak_ and VO_2Max_ should strongly correlate, particularly because participants reached VO2_Peak_ shortly before exercise failure. Energy Expenditure (EE) and Oxygen economy (OE), defined as oxygen intake per unit power output, were subsequently calculated using formulas from Moseley and Jeukendrup (2001).

### One-Minute Anaerobic Threshold Test

After the 80 rpm endurance exercise test, participants rested for approximately 5 min until their heart rates stabilized and returned to near baseline and they self-reported that they felt fully recovered. Participants then engaged in a 1-min anaerobic threshold test. This was included for two reasons. Firstly, to determine whether ketone supplementation could affect the maximum sustainable cadence PD subjects could maintain over a short period, which may reflect alterations in afferent stimulation of motor units (i.e., an altered capacity for the central nervous system to stimulate muscular contraction). Secondly, 1-min maximum intensity exercise is highly reliant on lactic glycolysis (Baker et al., 2010). Thus, we used this test to functionally assess whether KE supplementation might suppress glycolysis in PD subjects. Participants were instructed to cycle at as high a cadence as possible at their personalized wattage for 60 s. Cadence was recorded every second and average cadence was evaluated as the dependent variable. Immediate post-exercise (within 60 s) β-HB, glucose, and lactate levels were then measured. The exercise testing protocol, including both the endurance and anaerobic threshold tests, are shown in Figure 1.

### Statistical Analysis

Sample-size calculations were based on a report that a 12.6 rpm increase in cadence, from 66.0 to 78.6 rpm, translated into clinically meaningful differences in PD motor symptoms (Ridgel et al., 2015). Although our study instead explored the duration of time at which participants could hold this presumed-to-be-therapeutic ∼80 rpm cadence, these were the most relevant human data available upon which to base our calculation. To obtain 80% power with a significance level of 0.05, and allowing for 10% variance [6.6 rpm (Ridgel et al., 2015)] in participant performance, we calculated 12 participants would be required to detect a change in the primary outcome variable of endurance at 80 rpm. Two more participants were included to compensate for dropouts, bringing the total to 14 participants.

Blood data were analyzed using a two-way repeated measures ANOVA with Sidak corrections applied for multiple comparisons; respiratory measurements were analyzed using two-sided paired sample *t*-tests, and, because the performance data were not normally distributed, exercise performance was analyzed using Wilcoxon test. Differences were considered significant at *p* < 0.05. Values are reported as means ± standard error.

## Results

### Participant Characteristics

The ten male and four female participants who completed the study all had early stage PD. Mean disease duration was 4.9 years and no participant had disease advanced beyond Hoehn and Yahr stage 2. All were between the ages 48 and 71 years, non-obese, and did not possess diagnoses or injuries, other than PD, that would influence their capacity to exercise (Table 1).

### Blood β-Hydroxybutyrate, Glucose, and Lactate

The KE+CHO drink increased participants’ β-HB levels from 0.1 ± 0.0 to 3.5 ± 0.3 mmol/L within 30 min of consumption (Table 2). Following exercise, β-HB had decreased to 3.0 ± 0.3 mmol/L, reflecting the competing effects of β-HB oxidation by skeletal muscle during cycling (Cox et al., 2016) and the continued liberation of β-HB from the KE in the gut and liver (Stubbs et al., 2017). No increase in β-HB was observed in the CHO control group.

**TABLE 2:** Blood β-hydroxybutyrate, glucose, and lactate.

	Pre-drink		Pre-exercise (30 min After Drink)		Post-exercise	
	CHO	KE+CHO	CHO	KE+CHO	CHO	KE+CHO
β-HB (mmol/L)	0.1 ± 0.0	0.1 ± 0.0	0.1 ± 0.0	3.5 ± 0.3*	0.1 ± 0.0	3.0 ± 0.3*
Glucose (mmol/L)	4.8 ± 0.1	4.7 ± 0.1	8.8 ± 0.6	7.0 ± 0.4*	7.1 ± 0.8	4.3 ± 0.2*
Lactate (mmol/L)	3.2 ± 0.5	4.0 ± 0.6	2.8 ± 0.3	2.6 ± 0.4	8.5 ± 0.7	7.4 ± 0.6

Participants’ blood β-hydroxybutyrate (β-HB), glucose, and lactate levels ± standard error, as measured by fingerstick, prior to study drink ingestion, prior to exercise (30 min after study drink ingestion), and following exercise. *P*-values for between treatment differences were calculated using a two-way repeated measures ANOVA. * indicates *p* < 0.001 for the comparison between the carbohydrate (CHO) and ketone ester plus carbohydrate (KE+CHO) drinks at each timepoint. Data include *n* = 13 participants. One participant on a self-prescribed ketogenic diet with β-hydroxybutyrate levels >2 mmol/L was excluded from data summarized in this table because her data were an outlier and skewed the β-hydroxybutyrate values. However, inclusion or exclusion of this participant’s data from this or any data set presented in this study does not impact statistical significance.

Glucose rose by ∼1.7 mmol/L less in the KE+CHO group than in the CHO control (Table 2). While this could be explained simply be the fact that, as the calorie content of the drinks was the same, the control drink necessarily contained more carbohydrate, Mota AS et al. (manuscript under review) showed that KE decreases hepatic gluconeogenesis in patients with type 2 diabetics and that dysfunctional gluconeogenesis is largely responsible for poor glycemic control in type 2 diabetes, a common comorbidity of PD (Santiago et al., 2017).

### Ketone Ester Improved Endurance Performance and Did Not Affect Maximum Sustainable Cadence

**FIGURE 2:**
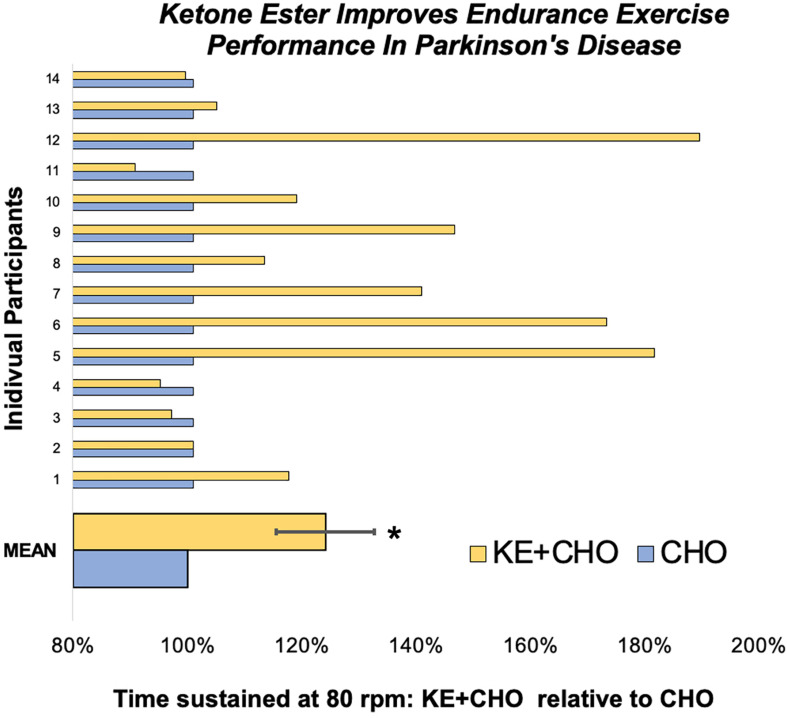
Endurance exercise test performance. On the endurance test, participant cycled for 24 ± 9% longer at 80 rpm following the ketone ester plus carbohydrate (KE+CHO) drink relative to performance following the isocaloric carbohydrate (CHO) drink. Data are expressed as performance following the KE+CHO drink divided by performance following the CHO drink, where performance is defined as the length of time participants could sustain 80 rpm. * indicates *p* = 0.027 as determined by Wilcoxon test.

Participants cycled for 24 ± 9% (*p* = 0.027) longer at the therapeutic target of 80 rpm (Ridgel et al., 2009, 2015) after consuming the KE+CHO drink compared to the isocaloric CHO control (Figure 2). Absolute time sustained on the KE+CHO and CHO arms were 13:46 ± 2:50 and 11:58 ± 2:43 min, respectively. The KE+CHO drink did not negatively impact maximum sustainable cadence, as measured on the 1-min anaerobic threshold test (absolute values: 113 ± 3 rpm on KE+CHO vs. 110 ± 4 rpm on CHO).

VO_2_ and VO_2Peak_ did not increase significantly (*p* = 0.08 and 0.07, respectively), whereas total V_E_ increased by >4 L/min. There was no change in VCO_2_, EE, and OE between the groups.

As expected, given that β-HB has a respiratory quotient (RQ) of 0.89 (Cox and Clarke, 2014) and increases intramuscular triglyceride (RQ = 0.70) oxidation (Cox et al., 2016), RER was significantly decreased in the KE+CHO arm relative to the CHO control arm, in which carbohydrate (RQ = 1.00) was the presumptive primary fuel. Mean rpm did not differ between the groups and so did not confound the results (Table 3).

**TABLE 3:** Respirometry data.

	RER	V_E_ (L/min)		VO_2_ (L/min)	VO_2Peak_ (L/min)
CHO	0.954 ± 0.012	47.42 ± 3.53		1.386 ± 0.121	1.81 ± 0.14
KE+CHO	0.931 ± 0.011	51.67 ± 4.52		1.502 ± 0.133	1.89 ± 0.15
*P*-value	0.03 ↓	0.03↑		0.08 ↑	0.07 ↑

	**VCO_2_ (L/min)**		**EE (kCal/hr)**	**OE (mL/Watt)**	**Cadence (rpm)**

CHO	1.410 ± 0.113		422.9 ± 35.5	0.30 ± 0.05	78.06 ± 0.78
KE+CHO	1.415 ± 0.129		450.0 ± 40.1	0.31 ± 0.04	77.76 ± 0.82
*P*-value	0.92		0.12	0.35	0.40

*Participants’ respiratory exchange ratio (RER), ventilation rate (V_*E*_), oxygen consumption rate (VO_2_), VO_2Peak_, carbon dioxide expiration rate (VCO_2_), energy expenditure (EE), oxygen economy (OE), and mean revolutions per minute (rpm) ± standard error on the endurance exercise test. *P*-values were calculated using two-tailed paired sample *t*-tests and arrows indicate the significant or trending directional effects of the ketone ester plus carbohydrate (KE+CHO) drink relative to the isocaloric carbohydrate (CHO) control.*

## Discussion

### Ketone-Induced Improvements in Endurance

Consumption of a KE+CHO nutritional supplement increased, by 24 ± 9%, the time individuals with PD sustained an 80 rpm cycling cadence, relative to their performance following an isocaloric CHO control drink. At a systems level, the associated changes, or lack thereof, in our secondary outcome variables (in particular, β-HB, RER, VO_2_, VO_2Peak_, VCO_2_, and lactate) were consistent with those thought to enhance endurance performance in athletes.

As expected, a single KE drink significantly increased β-HB to >3 mmol/L, ketosis equivalent to that observed after several days of fasting and one difficult to achieve even on the strictest ketogenic diet. The ketotic state achieved by the study participants explains the observed decrease in RER. Compared to CHO, which has an RQ of 1.00, β-HB has an RQ of 0.89 (Cox and Clarke, 2014). Furthermore, ketosis induced by KE+CHO drink ingestion increases the oxidation of intramuscular triglycerides (RQ of 0.70) during endurance exercise (Cox et al., 2016). Thus, β-HB oxidation and a shift to fat catabolism can explain the decrease in RER.

As RER is defined as the ratio VCO_2_/VO_2_ [and more O_2_ is oxidized during fat oxidation compared to glucose oxidization because fatty acids are a more reduced substrate (Cox and Clarke, 2014)], it makes sense that we observed a trend toward increasing VO_2_ without any trend in VCO_2_. Concordant with VO_2_, VO_2Peak_ too trended upward in the KE+CHO group relative to the control, although did not achieve statistical significance, likely because of the limited sample size. Nevertheless, the putative 80 mL/min increase in VO_2Peak_ is notable because VO_2Max_ (see “Methods” for distinction) is a marker of cardiovascular fitness, one of the best predictors of longevity (Ladenvall et al., 2016), and has been associated with elevated cognitive and motor scores in PD (Uc et al., 2008). Admittedly, VO_2Peak_, as measured in this acute intervention study, did not reflect an improvement in participants’ overall fitness; however, by increasing the ability of persons with PD to sustain endurance exercise, a KE supplement could improve fitness and true VO_2Max_. An interesting qualification to this prediction is that persons with PD exhibit impaired metabolic adaptations in VO_2Max_ and V_E_ in response to routine aerobic exercise (Mavrommati et al., 2017). Therefore, it’s possible that increasing endurance may not translate into improved fitness even with training. Alternatively, it’s also possible that KE could help to compensate for systemic metabolic dysfunction in persons with PD to rescue their ability to adapt to aerobic exercise (see discussion of β-oxidation insufficiency and CI blockade below).

**FIGURE 3:**
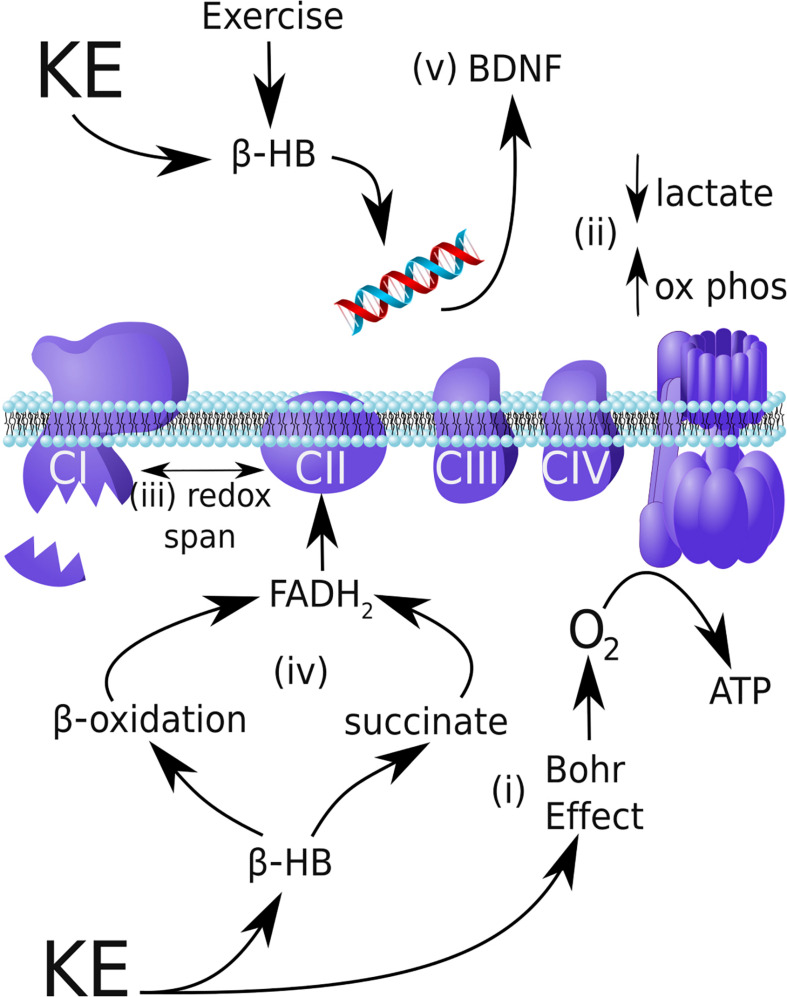
Possible mechanisms by which a ketone ester can improve endurance performance and provide neuroprotection in Parkinson’s disease. **(i)** By safely shifting blood pH to induce the Bohr Effect, the ketone ester (KE) supplement can increase oxygen delivery to skeletal muscle and increase VO_2_. **(ii)** β-HB shifts exercise metabolism toward mitochondrial oxidative phosphorylation during exercise relative to glycolysis, reducing lactate production. **(iii)** In mitochondria, β-HB can increase the redox span of the respiratory chain, thereby increasing the efficiency of energy production by oxidative metabolism. **(iv)** In Parkinson’s disease, complex I (CI) is damaged and underactive, whereas complex II (CII) is entirely functional. By increasing β-oxidation of intramuscular triglycerides and increasing succinate, β-HB can increase CII flux and circumvent the CI blockade. **(v)** In addition to reducing oxidative stress and inflammation (not shown), β-HB produced during exercise inhibits HDACs and activates CREB/CRP signaling to upregulate neuroprotective BDNF expression.

The lack of change in VCO_2_, despite an increase in overall V_E_, is itself notable when one considers the metabolism of the KE drink. The butanediol component of the KE is metabolized in the liver to form a keto-acid, which lowers blood pH from 7.41 to 7.31 within 1 h of KE ingestion (Stubbs et al., 2017). As blood acidification drives a rightward shift in the oxygen-hemoglobin dissociation curve via the Bohr effect, the moderate acidification could improve the efficiency of O_2_ unloading at skeletal muscles and contribute to an increase in VO_2_ and VO_2Peak_ (Malte and Lykkeboe, 2018; Figure 3i). Blood acidification and O_2_ unloading also increase the affinity of hemoglobin for CO_2_ by the Haldane effect (the converse of the Bohr effect), which increases CO_2_ binding to hemoglobin and carbaminohemoglobin formation (Feher, 2017). Furthermore, blood acidification inhibits carbonic anhydrase activity (Berg et al., 2002). This predicts that KE ingestion could cause mild CO_2_ sequestration during exercise, perhaps driving up V_E_.

The trend to lower lactate levels post-exercise following KE+CHO ingestion, as compared to CHO ingestion, is also worth remarking upon in the context of the literature. Lactate levels were higher at baseline in our participants than one would expect of individuals without PD, which is consistent with research demonstrating ∼50% elevated resting lactate in patients with PD and metabolically similar conditions (multiple sclerosis and mitochondrial complex I (CI) deficiency) relative to healthy controls (Landin et al., 1974; Roef et al., 2002; Keytsman et al., 2019). The high baseline lactate may be explained by the 16–54% decreased peripheral CI activity in PD patients (Parker et al., 1989; Krige et al., 1992; Yoshino et al., 1992; Benecke et al., 1993; Haas et al., 1995; Blake et al., 1997; Petrus et al., 2019), including in skeletal muscle (Bindoff et al., 1991; Blin et al., 1994). We refer to this as the “CI blockade.” Impaired mitochondrial respiration would force a relative shift toward glycolysis, generating more lactate. KE+CHO supplementation in elite athletes diminishes lactate accumulation during exercise by ∼50% by shifting metabolism from glycolysis toward oxidative phosphorylation (Cox et al., 2016; Figure 3ii). Consistent with these prior findings, in the present study we observed the same trend toward a blunted lactate spike following the KE+CHO drink.

The changes in β-HB and RER (as well as the noted trends in VO_2_, VO_2Peak_, and lactate, for which this study was not sufficiently powered nor was it intended to be) are consistent with the literature, and predict that the KE+CHO drink would improve endurance exercise performance in PD, as we observed. However, the above-mentioned mechanisms may not fully explain the 24% effect size.

### Mitochondrial Efficiency and Enhanced Endurance

A general cellular explanation for how the KE+CHO drink improved endurance performance in our participants is that the β-HB component of the drink improved mitochondrial efficiency. β-HB has the potential to increase ATP production, and/or the free energy liberated by ATP hydrolysis, by exerting opposite redox effects on the respiratory chain electron carriers, NADH and CoQ10 (Sato et al., 1995). This mechanism of increasing redox potential energy within the mitochondrial respiratory chain is analogous to increasing gravitational potential energy by lifting a bowling ball “electron” higher from the ground (Norwitz et al., 2019). Stated concisely, ketosis may increase the redox span of the electron transport chain to increase the efficiency of mitochondrial metabolism (Figure 3iii). This mechanism is relevant to, but not specific to, PD.

PD is characterized by an increased dependence on carbohydrate, relative to fat, as fuel. Early work by Landin et al. (1974) showed that individuals with PD deplete glycogen during exercise at four-fold the rate of control subjects and exhibit decreased fatty acid turnover. This impaired fatty acid metabolism has been confirmed more recently by Saiki et al., who showed that β-oxidation is impaired early in PD, independent of levodopa dose. They termed this phenomenon Parkinson’s “β-oxidation insufficiency” (Saiki et al., 2017). We have shown previously that KE shifts metabolism to increase the β-oxidation of intramuscular triglycerides in athletes (Cox et al., 2016). If this phenomenon is generalizable to PD, a correction of “β-oxidation insufficiency” could contribute to the large effect size of KE on endurance performance reported in this study.

Furthermore, as mentioned above, PD is metabolically characterized by the mitochondrial “CI blockade.” CI dysfunction is not simply localized to the brain, but extends to the periphery, including to skeletal muscle (Parker et al., 1989; Bindoff et al., 1991; Krige et al., 1992; Yoshino et al., 1992; Benecke et al., 1993; Blin et al., 1994; Haas et al., 1995; Blake et al., 1997; Petrus et al., 2019). As electrons can enter the respiratory chain at either CI or complex II (CII), and CII function does not appear to be impaired in PD (Blin et al., 1994; Tieu et al., 2003), interventions that promote CII-flux over CI-flux would be predicted to enhance mitochondrial metabolism in PD.

By increasing β-oxidation and also by increasing succinate flux, as compared to glucose, the KE drink provided to participants in this study should preferentially increase CII activity to improve mitochondrial metabolism in PD (Figure 3iv). With regard to β-oxidation, each cycle of β-oxidation generates 1 FADH_2_: 1 NADH, as compared to each Krebs cycle, which generates 1 FADH_2_: 3 NADH. Thus, the oxidation of fatty acids by β-oxidation and the Krebs cycle generates a higher FADH_2_: NADH ratio than the oxidation of glucose and shifts electron flux towards CII. (As an example, the oxidation of C18:0 stearic acid, through eight cycles of β-oxidation and nine Krebs cycles, generates a FADH_2_: NADH ratio of 16:32 or 0.5, whereas oxidation of glucose yields an FADH_2_: NADH ratio of 2:10 or 0.2). With regard to succinate flux, the rate limiting step of β-HB catabolism generates succinate, which is used to reduce FAD to FADH_2_; and, thus, succinate serves as an oxidative fuel for CII. Beyond mechanistic speculation, Tieu et al. (2003) showed that administration of β-HB to MPTP-treated PD mice protected dopaminergic SNpc neurons from cell death and that this effect is blocked by the specific inhibition of CII.

There is, therefore, a mechanistic and animal model basis for hypothesizing that KE improves endurance performance in PD, specifically, by helping to correct “β-oxidation insufficiency” and circumvent the “CI blockade.”

### Ketone Ester Holds Promise as an Indirect Disease-Modifying Therapy for Parkinson’s Disease

By improving patients’ abilities to engage in therapeutic 80 rpm cycling, KE could indirectly provide disease-modifying neuroprotection in PD. Earlier, we mentioned exercise paradox for PD: vigorous exercise may slow disease progression, but as the disease progresses patients lose the ability to engage in vigorous exercise. If our data are replicated in larger and longer-term studies, patients of the future could supplement with KE prior to exercise in order to engage in more vigorous more effective exercise over longer periods of time. KE supplementation could eliminate, or at least help compensate for, the exercise paradox, thereby providing patients with a tool to improve their disease trajectory.

### Ketone Ester Holds Promise as a Direct Disease-Modifying Therapy for Parkinson’s Disease and May Synergize With Exercise

This study was an acute intervention that focused on using KE as an adjunct to boost the efficacy of exercise therapy. However, it is important to note that the β-HB molecule, upon which KE is based, has direct disease-modifying potential in PD. As we have previously described how β-HB can address the foundational metabolic dysfunctions that underly to PD: energetic dysfunctions, oxidative stress, inflammation, and cell death (Norwitz et al., 2019), we here specifically elaborate on how β-HB and exercise may work together to protect against and slow the progression of PD, through mechanisms involving neurotrophic factors.

Work on animal models of PD has demonstrated that part of the neuroprotection afforded by endurance exercise is attributable to BDNF and GDNF, two neurotrophic factors that are reduced in the SNpc of human PD patients by as much as 20% per neuron, relative to healthy age-matched controls (Chauhan et al., 2001). In independent studies, aerobic exercise protected rats from motor deficits induced by direct injection of 6-OHDA into the striatum. The preservation of motor function in the exercised rats was associated with roughly 60% increases in BDNF and GDNF, and further with the preservation of dopaminergic SNpc neurons (Cohen et al., 2003; Tajiri et al., 2010). Other studies using MPTP rodent models of PD support these findings (Tillerson et al., 2003; Pothakos et al., 2009; Gerecke et al., 2010) and provide encouragement that exercise is neuroprotective in PD.

The BDNF/GNDF neuroprotective mechanism directly implicates β-HB. *In vitro*, β-HB induces neuronal BDNF expression (Marosi et al., 2016; Kim et al., 2017), and inhibition of the BDNF receptor diminishes the neuroprotective benefits of β-HB (Kim et al., 2017). *In vivo*, in mice, 6 weeks of exercise increased β-HB and BDNF levels in the brain (Marosi et al., 2016). Other data validate and elaborate upon this “exercise-β-HB-BDNF/GNDF” model. Sleiman et al. (2016) found that exercise increased β-HB in mice and that β-HB induces BDNF in primary cortical neurons by acting as a histone deacetylase (HDAC) inhibitor and decreasing HDAC binding to the BDNF promotor. What’s more, exercise and direct intraventricular delivery of β-HB were each sufficient to increase BDNF expression in the brain (Sleiman et al., 2016).

D-β-hydroxybutyrate appears to act more generally as an epigenetic modifier to increase BDNF expression. Not only does β-HB block histone deacetylation to increase BDNF expression, but Hu et al. (2018) showed that β-HB actively upregulates BDNF promoter acetylation by inducing cAMP response element-binding protein (CREB)/CREB-binding protein (CBP) signaling. Therefore, by the complementary mechanisms of downregulation of deacetylation and upregulation of acetylation [along with other putative mechanisms such as inducing histone demethylation (Hu et al., 2018) and adaptive hormetic oxidative stress (Kim et al., 2017)], β-HB can increase BDNF expression to support neuron survival (Figure 3v).

Critically, in the *in vivo* studies conducted by Hu et al. (2018) the dose of β-HB administer to mice (120 mg/kg/day) that increased BDNF expression was 18-times less than the dose of ΔG^®^ KE drink that has been validated as safe in humans (2.142 g/kg/day) (Clarke et al., 2012). Therefore, there is at least early animal evidence in support of the notion that exogenous ketone supplements, and specifically the ΔG^®^ KE drink used in this study, would exert neuroprotective benefits in PD, not only indirectly, by increasing exercise performance, but also directly, by increasing BDNF. This is an area ripe for future investigations.

### Limitations

Our study has several limitations. First, PD symptomology is highly variable day to day. Therefore, although our crossover design allowed participants to serve as their own controls, intrapersonal variability was an unavoidable complicating factor. Second, we chose to test participants in an “off” medication state so as to isolate the impact of the intervention. As the KE supplement holds potential primarily as an adjunct to, and not a replacement for, dopaminergic therapy, future studies should examine the impact of the KE drink when patients are “on” medications. Third, while our study was sufficiently powered to achieve statistical significance with respect to the main outcome variable, a larger sample size may have allowed us to report significant differences for VO_2_, VO_2Peak_, and lactate, which are consistent with previous literature and are relevant to the mechanistic understanding of why the KE+CHO drink enhanced performance. Fourth, this study was aimed only at investigating the acute effects of ketosis on exercise performance in PD, and therefore excludes the potentially larger neuroprotective benefits derived from chronic ketosis.

### Future Directions

Larger scale studies are required to validate these results. Future studies should examine the mechanisms of any effect, specifically the possibility that the KE corrects Parkinson’s “β-oxidation insufficiency” and compensates for the “CI blockade.” Finally, it would be invaluable to conduct rigorous large-scale prospective studies on the hypothesized protective benefits of ketogenic interventions in the coming decades in order to properly assess whether exercise and ketosis, independently and together, can be used as true disease-modifying treatments for PD.

## Data Availability Statement

The raw data supporting the conclusions of this article will be made available by the authors, without undue reservation.

## Ethics Statement

The studies involving human participants were reviewed and approved by the National Health Service Health Research Authority & South Central – Oxford A Research Ethics Committee, REC reference: 19/SC/0032. The patients/participants provided their written informed consent to participate in this study.

## Author Contributions

All authors have contributed to this work and approved it for publication.

## Conflict of Interest

KC is inventor of the KE used in this study and director of TΔS Ltd., the company produces the KE. Intellectual property covering uses KE supplementation is jointly owned by TΔS Ltd., BTG Ltd., the University of Oxford, and the National Institutes of Health. Should royalties ever accrue from these patents, KC, as inventor, will receive a share of the royalties under the terms prescribed by the University of Oxford. KC is a director of TΔS Ltd., a company spun out of the University of Oxford to develop and commercialize products based on the science of ketone bodies in human nutrition. The remaining authors declare that the research was conducted in the absence of any commercial or financial relationships that could be construed as a potential conflict of interest.

## Abbreviations

BDNF: brain-derived neurotrophic factor
β-HB: D- β-hydroxybutyrate
CBP: CREB-binding protein
CHO: carbohydrate
CoQ10: coenzyme Q10
CREB: cAMP response element-binding protein
EE: energy expenditure
FADH_2_: flavin adenine dinucleotide
GDNF: glial-derived neurotrophic factor
HDAC: histone deacetylase
KE: ketone ester
LEDD: levodopa equivalent daily dose
MDS-UPDRS: Movement Disorder Society-Unified Parkinson’s Disease Ratings Scale
MPTP: 1-methyl-4-phenyl-1,2,3,6-tetrahydropyridine
NADH: nicotinamide adenine dinucleotide
OE: oxygen economy
PD: Parkinson’s disease
RER: respiratory exchange ratio
RPE: relative perceived exertion
rpm: revolutions per minute
RQ: respiratory quotient
SNpc: substantia nigra pars compacta
SPARX: Study in Parkinson’s Disease of Exercise
UPDRS: Unified Parkinson’s Disease Ratings Scale
VCO_2_: carbon dioxide expiration rate
V_E_: ventilation rate
VEGF: vascular endothelial growth factor
VO_2_: oxygen consumption rate
VO_2Max_: maximum oxygen consumption rate
VO_2Peak_: peak oxygen consumption rate on endurance test
6-OHDA: 6-hydroxydopamine
Δ G^®^: (R)-3-hydroxybutyl (R)-3-hydroxybutyrate ketone monoester.
